# One‐Step Method for Instant Generation of Advanced Allogeneic NK Cells

**DOI:** 10.1002/advs.201800447

**Published:** 2018-09-27

**Authors:** Daniel Y. Lee, Kwang Suk Lim, Gabriel M. Valencia, Minjin Jung, David A. Bull, Young‐Wook Won

**Affiliations:** ^1^ Division of Cardio‐Thoracic Surgery Department of Surgery University of Utah School of Medicine Salt Lake City UT 84132 USA; ^2^ Division of Cardio‐Thoracic Surgery Department of Surgery University of Arizona College of Medicine Tucson AZ 85724 USA; ^3^ Department of Biotechnology and Bioengineering Institute of Forest Science Kangwon National University Chuncheon‐si 24341 Republic of Korea

**Keywords:** antibody–drug conjugates, cancer, chemoimmunotherapy, immune cells, one‐step method

## Abstract

Conventional combinatorial anticancer therapy has shown promising outcomes; still, a significant interest in developing new methods to reinforce and possibly merge chemotherapy and immunotherapy persists. Here, a new one‐step method that immediately modifies immune cells into a targeted form of chemoimmunotherapy through spontaneous and rapid incorporation of hydrophobized antibody–drug conjugates (ADCs) on the surface of immune cells is presented. Therapeutic objectives of this approach include targeted delivery of a potent chemotherapeutic agent to avoid adverse effects, enhancing the mobilization of infused immune cells toward tumor sites, and preserving the intense cytotoxic activities of immune cells against tumor cells. The embedding of hydrophobized ADCs on the immune cell membrane using the strategy in this study provides noninvasive, nontoxic, and homogenous modifications that transiently arm immune cells with highly potent cytotoxic drugs targeted toward cancer cells. The resulting surface‐engineered immune cells with ADCs significantly suppress the tumor growth and drive the eradication of target cancer cells through combinatorial anticancer effects. This novel strategy allows convenient and timely preparation of advanced chemoimmunotherapy on a single immune cell to treat various types of cancer.

## 1 Introduction

Advanced cancer therapies have recently focused on combining chemotherapy and immunotherapy to promote therapeutically beneficial synergy for maximizing the clinical antitumor activity.^1^, ^2^ Effective chemoimmunotherapy requires that (1) chemotherapeutic agents induce cancer cell death and promote immunomodulation, (2) targeted chemotherapy minimizes the adverse effects on immune cells, and (3) immune effector cells maintain their cytolytic activity against cancer cells.^1^, ^3^, ^4^ New strategies to combine chemotherapy and immunotherapy that meet the above criteria would represent a new platform for the development of targeted cancer chemoimmunotherapy.

Monoclonal antibodies (mAbs) have been established as the mainstream mode of immunotherapy in clinical oncology as well as excellent vehicles for targeted delivery of cytotoxic agents.^5^, ^6^, ^7^ Over the past two decades, antibody–drug conjugates (ADCs) were developed and continued to show promising clinical responses through increasing the payload of highly potent anticancer drugs and overcoming the off‐target adverse effects.^8^ In company with ADCs, immune cells emerged as an innovative immunotherapy as the technologies in isolating tumor‐active immune cells from a patient and growing them into sufficient numbers for reinfusion in ex vivo condition started to develop.^9^ Genetic engineering enabled the customization of receptors on immune cells to target specific cancer antigens and the resulting chimeric antigen receptor T (CAR‐T) cells have received clinical attention and support.^10^ Although ADCs and immune cell therapies are the state‐of‐the‐art technologies, the synergy between ADCs and immune cell therapies has become attractive to achieve greater clinical anticancer response. In this combinatorial approach, ADCs induce immunogenic cancer cell death that makes the dying cancer cells much more obvious for transferred immune cells to eliminate.^11^ To attain this potential therapeutic benefit, it is necessary to invite creative methods that support the concurrent delivery of ADCs and immune cells to the tumor site.

We developed a one‐step method that spontaneously transforms nonspecific immune cells into a new form of targeted chemoimmunotherapy through the introduction of ADCs on the surface of an active immune cell. The process of embedding ADCs on the immune cell surface exploits the hydrophobic interaction between a polymeric lipid chain and the lipid bilayer of the cell membrane. Likewise, lipid‐conjugated ADCs (hydrophobized ADCs) can be readily incorporated into the lipid bilayer without disrupting the cell membrane integrity and award new functions to the surface‐engineered cells.^12^, ^13^ We hypothesized that the surface‐engineered immune cells containing ADCs could simultaneously deliver potent chemotherapeutic agents to the target tumor and enhance the homing of adoptively transferred immune cells toward the tumor sites without compromising their cytotoxic activities, ultimately intensifying the combinatorial anticancer efficacy to combat cancer.

**Figure 1:**
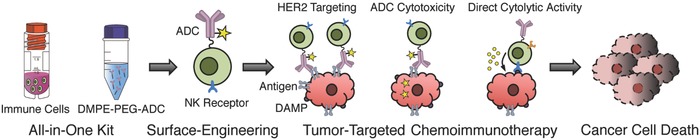
Illustration showing the framework of targeted chemoimmunotherapy generated using our one‐step method. The DMPE‐PEG‐ADC is prepared as a ready‐to‐use formulation and pre‐expanded immune cells are transformed to a targeted form of chemoimmunotherapy by mixing immune cells and DMPE‐PEG‐ADC together for 15 min. Immune cells equipped with ADCs migrate toward the target tumor site through the recognition of target antigen by ADCs. In the target tumor tissues, ADCs induce apoptosis of the target cancer cells and immune cells present in proximity destroy the dying cancer cells that expressed damage‐associated molecular patterns (DAMP).

To prove our hypothesis, trastuzumab emtansine (T‐DM1), a model ADC, was first modified by attaching 1,2‐dimyristoyl‐sn‐glycero‐3‐phosphoethanolamine (DMPE) conjugated polyethylene glycol (PEG) and the resulting hydrophobized T‐DM1 (DMPE‐PEG‐T‐DM1) was used to modify the surface of allogeneic natural killer (NK) cells, a model immune cell. These T‐DM1 surface‐engineered NK (SE‐NK/T‐DM1) cells, the product generated using our approach, recognized and destroyed human epidermal growth factor receptor 2 (HER2)‐positive cancer cells through the combined activity of T‐DM1 and NK cells. This single‐injection formulation chemoimmunotherapy, SE‐NK/T‐DM1 cells, suppressed the progression of the target tumor significantly compared to the cotreatment of NK cells and T‐DM1. Our innovative strategy to instantaneously generate advanced immune cells as “off‐the‐shelf” chemoimmunotherapeutic reagents is therapeutically advantageous over the conventional chemoimmunotherapeutic strategies because it simultaneously delivers antibodies, cytotoxic agents, and immune effector cells to the target tumor (**Figure** **1**).

## 2 Results and Discussion

**Figure 2:**
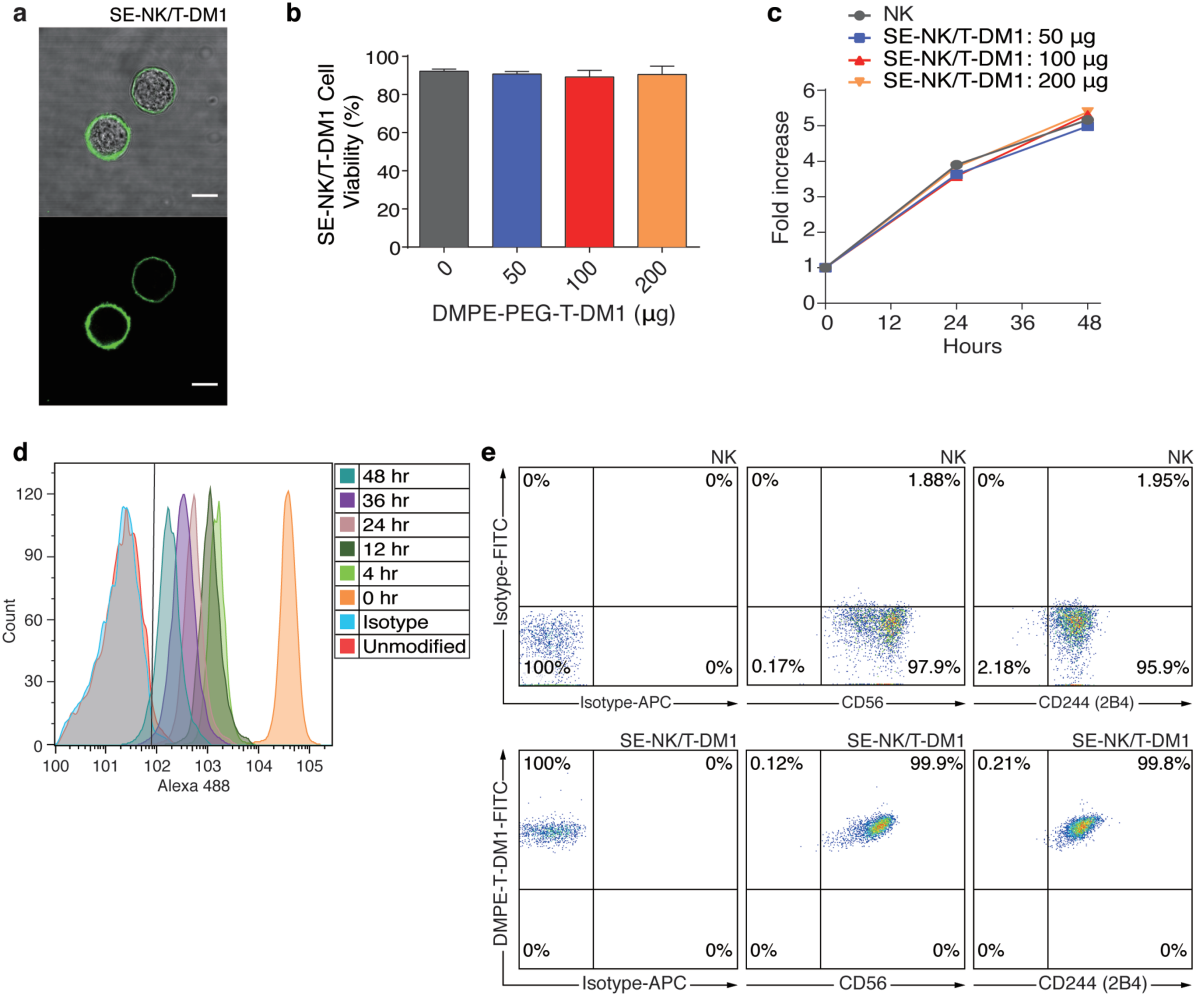
One‐step method reliably generates immune cells equipped with ADCs without disturbing the cellular functions and viability. a) Confocal microscopy image of SE‐NK/T‐DM1 cells generated with 100 µg of FITC‐labeled DMPE‐PEG‐T‐DM1 (scale bars: 10 µm). b) Comparison of cell viabilities between unmodified NK cells and SE‐NK/T‐DM1 cells generated with 50, 100, or 200 µg of DMPE‐PEG‐T‐DM1. Data represent mean ± SD. c) Comparison of cell proliferation between unmodified NK cells and SE‐NK/T‐DM1 cells generated with 50, 100, or 200 µg of DMPE‐PEG‐T‐DM1. Data represent mean ± SD. d) Surface retention time of T‐DM1 on the surface of SE‐NK/T‐DM1 cells incubated in media containing 10% serum. SE‐NK/T‐DM1 cells were incubated in complete growth media and a portion of cells were sampled at each time point up to 48 h. The presence of T‐DM1 on the surface of SE‐NK/T‐DM1 cells was detected using Alexa 488‐conjugated goat anti‐human IgG (H+L) antibodies. Plot is selected from two independent experiments. e) Availability of CD56 and 2B4 on the cell membrane of SE‐NK/T‐DM1‐FITC cells was determined by flow cytometry. After SE‐NK/T‐DM1‐FITC cells were prepared, APC‐conjugated anti‐CD56 antibodies or APC‐conjugated anti‐2B4 antibodies were used to detect the receptor availability. All images and plots are from one of two independent experiments.

T‐DM1 was generated through the expression of trastuzumab (TZ) in mammalian cells followed by DM1 conjugations.^14^, ^15^ Prepared T‐DM1 was subsequently hydrophobized by attaching DMPE‐PEG‐NHS, resulting in the production of DMPE‐PEG‐T‐DM1 (Figure S1, Supporting Information). T‐DM1 synthesized in our lab exhibited similar cytotoxicity compared to the commercial product, Kadcyla (Figure S2, Supporting Information). Surface engineering of NK cells with various amounts of DMPE‐PEG‐T‐DM1 affects neither the viability nor the proliferative activity of NK cells (**Figure** **2**a–c). Reliable modification of 5 × 10^5^ immune cells required 100 µg of DMPE‐PEG‐T‐DM1 that yielded ≈2.1 µg of T‐DM1 embedded on the cell membrane of 1 × 10^5^ SE‐NK/T‐DM1 cells (Figure S3, Supporting Information).

T‐DM1 was detected on the surface of SE‐NK/T‐DM1 cells for over 48 h in complete growth media (Figure 2d) and two of the key NK cell‐specific markers, CD56 and 2B4, were available on the surface of SE‐NK/T‐DM1 cells. These results demonstrate that T‐DM1 is embedded on the NK cell surface without internalization and the surface engineering of NK cells with ADCs does not interfere with the NK cell receptor accessibility, suggesting that the inherent cytolytic activity of NK cells is retained upon the surface engineering (Figure 2e). Because the allogeneic NK cells used in this study, NK‐92 cells, lack CD16, CD32, and CD64 IgG receptors that can initiate antibody internalization and antibody‐dependent cellular cytotoxicity (ADCC),^16^ the surface engineering with T‐DM1 appears to have minimal effects on NK cell metabolism and viability. In cancer cells, T‐DM1 internalization occurs through HER2 receptor‐mediated endocytosis.^17^ NK cells do not express HER2 on their membrane, therefore DMPE‐PEG‐T‐DM1 embedded on the surface of SE‐NK/T‐DM1 cells were not internalized and showed negligible cytotoxicity. Moreover, PEG spacer between the DMEP and T‐DM1 provides a physical barrier for internalization.^18^ Studies have reported that longer PEG spacers not only inhibit the internalization of biomolecules but also reduce the membrane insertion efficiency by increasing the steric hindrance.^18^, ^19^, ^20^ The aforementioned membrane insertion efficiency of DMPE‐PEG‐T‐DM1 yielded about 10% due to the presence of long PEG spacer (Figure S3, Supporting Information).

**Figure 3:**
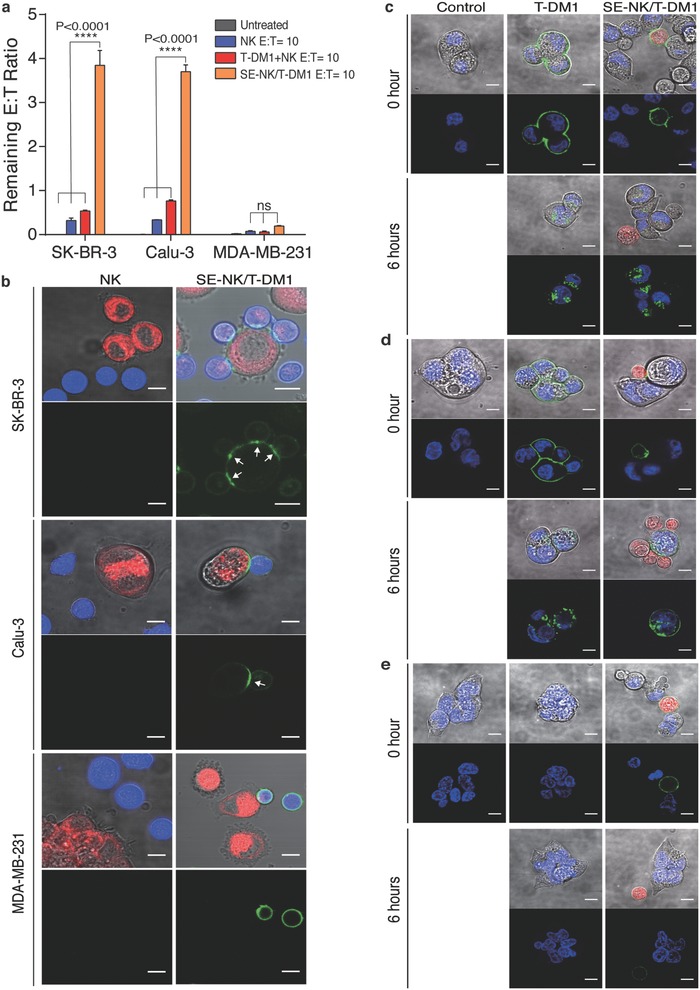
ADCs embedded on the cell surface deliver the immune cells toward the target cancer cells then transfer and internalize into the target cancer cells. a) Binding of SE‐NK/T‐DM1 cells to the HER2‐positive cancer cells. Cancer cells were coincubated with NK cells, SE‐NK/T‐DM1 cells, or T‐DM1+NK cotreatment at an *E*:*T* ratio of 10:1. After 30 min, unbound cells were thoroughly washed and the remaining NK cells were counted using flow cytometry to calculate the remaining *E*:*T* ratio. Cancer cells were labeled in red with CellTracker Red CMTPX and NK cells were labeled in blue with CellTracker Blue CMAC. Data represent mean ± SD (ns, not significant; ****P < 0.0001, by one‐way ANOVA with Bonferroni post hoc tests). b) Confocal microscopy images showing the binding of SE‐NK/T‐DM1 cells and transfer of T‐DM1 from SE‐NK/T‐DM1 cells to SK‐BR‐3 cells, Calu‐3 cells, and MDA‐MB‐231 cells. Cancer cells (red) were coincubated with NK cells (blue) or SE‐NK/T‐DM‐FITC cells (NK cells in blue and T‐DM1 in green) at an *E*:*T* ratio of 10:1. Unbound effector cells were thoroughly washed after 30 min of coincubation and the remaining cells were observed in live by confocal microscopy. Polarization of T‐DM1‐FITC (green) at the effector cell‐to‐target cell junction is indicated with white arrows. DMPE‐PEG‐T‐DM1 was able to move across the SE‐NK/T‐DM1 cell membrane to the contact point and formed antigen–antibody complexes with HER2 expressed on cancer cells. Subsequently, the antigen–antibody complexes spread across the cancer cell membrane through membrane fluidity. Scale bars: 10 µm. All data are representative of two independent experiments. c–e) Internalization of transferred T‐DM1 into HER2‐positive SK‐BR‐3 cells, HER2‐positivie Calu‐3 cells, and HER2‐negative MDA‐Mb‐231. Cancer cells labeled with nuclear staining dye (blue) were seeded on an eight‐chambered cover glass slide and incubated SE‐NK/T‐DM1‐FITC cells (NK cells in red, T‐DM1 in green) at an *E*:*T* ratio of 10:1. For a comparison, FITC‐labeled T‐DM1 (green) was treated to each cancer cells. Unbound NK cells were thoroughly removed after 30 min of incubation and the remaining cancer cell‐bound NK cells were imaged by confocal microscopy to detect internalized T‐DM1 in the cancer cell cytoplasm. Images were taken at the initial time point of treatment and 6 h later. Scale bars: 10 µm. All data are representative of two independent experiments.

To demonstrate the specific binding of SE‐NK/T‐DM1 cells to their target cancer cells, the number of remaining NK cells was determined after coincubating SE‐NK/T‐DM1 cells with the target cancer cells or the nontarget cancer cells. Unmodified NK cells, T‐DM1 and NK cells (T‐DM1+NK) cotreatment, or SE‐NK/T‐DM1 cells were incubated with HER2‐positive SK‐BR‐3 cells, HER2‐positive Calu‐3 cells, or HER2‐negative MDA‐MB‐231 cells at an effector‐to‐target (*E*:*T*) ratio of 10:1 (**Figure** **3**a). After 30 min of coincubation, unbound NK cells were thoroughly washed and the remaining cells were counted using flow cytometry. The remaining *E*:*T* ratios when cancer cells were treated with SE‐NK/T‐DM1 cells, T‐DM1+NK cotreatment, and unmodified NK cells were ≈3.8, 0.5, and 0.3 on SK‐BR‐3 cells; and, 3.7, 0.8, and 0.3 on Calu‐3 cells, respectively. Negligible numbers of NK cells remained bound to MDA‐MB‐231 cells. These results revealed that SE‐NK/T‐DM1 cells specifically recognize and bind to HER2‐positive cancer cells.

In order for T‐DM1 to exert its anticancer activity on cancer cells, T‐DM1 on the SE‐NK/T‐DM1 cells must transfer to the target cancer cells. We coincubated unmodified NK cells and SE‐NK/T‐DM1‐FITC cells with SK‐BR‐3 cells, Calu‐3 cells, or MDA‐MB‐231 cells on an eight‐chambered cover glass sides. Unbound NK cells were removed after 30 min and the transfer of T‐DM1‐FITC was observed through confocal microscopy. Upon the binding of SE‐NK/T‐DM1 cells to SK‐BR‐3 cells and Calu‐3 cells (Figure 3b, top and middle), T‐DM1 migrated toward the contact area, formed clusters at the effector cell‐to‐cancer cell junction (Figure 3b, white arrows), and subsequently transferred onto the target cancer cells. Lipids contained in DMPE‐PEG‐T‐DM1 allow the lateral movement of T‐DM1 across the NK cell membrane.^13^ Through this attribute, DMPE‐PEG‐T‐DM1 was able to polarize toward the contact point between NK cells and cancer cells where HER2 is presented. Once DMPE‐PEG‐T‐DM1 binds to HER2 on cancer cells, these antigen–antibody complexes spread across the cancer cell membrane following lateral movement of HER2 on cancer cells. Polarization of DMPE‐PEG‐T‐DM1, formation of antigen–antibody complex, and transfer of DMPE‐PEG‐T‐DM1 were missing when SE‐NK/T‐DM1 cells were treated to HER2‐negative MDA‐MB‐231 cells (Figure 3b, bottom). These results indicate that T‐DM1 embedded on the surface of NK cells relocates onto the target cancer cell membrane after forming the antigen–antibody complex.

Internalization of T‐DM1 is crucial for its anticancer efficacy because DM1 acts on intracellular targets in cancer cells.^7^, ^21^ In keeping with previously reported observations on cellular uptake of T‐DM1, trafficking of T‐DM1 to lysosomes, and release of DM1,^22^ we focused on confirming the internalization of T‐DM1 transferred from SE‐NK/T‐DM1 cells in the target cancer cells. Cancer cells plated on an eight‐chambered cover glass slide were labeled with nuclear stating dye (blue) to observe the location of FITC‐labeled T‐DM1 (green) transferred from SE‐NK/T‐DM1‐FITC cells (red). Identical to the study above, unbound NK cells were thoroughly removed after 30 min of coincubation. Distinct fluorescent dots representing the internalized T‐DM1, following the transfer from the assessed SE‐NK/T‐DM1 cells, were detected in the cytoplasm of target cancer cells (Figure 3c,d), representing the internalization of T‐DM1 into the target cancer cells. No fluorescent activity was observed in MDA‐MB‐231 cells treated with the identical conditions used in HER2‐positive cancer cells (Figure 3e), confirming no internalization of T‐DM1 in HER2‐negative cancer cells.

**Figure 4:**
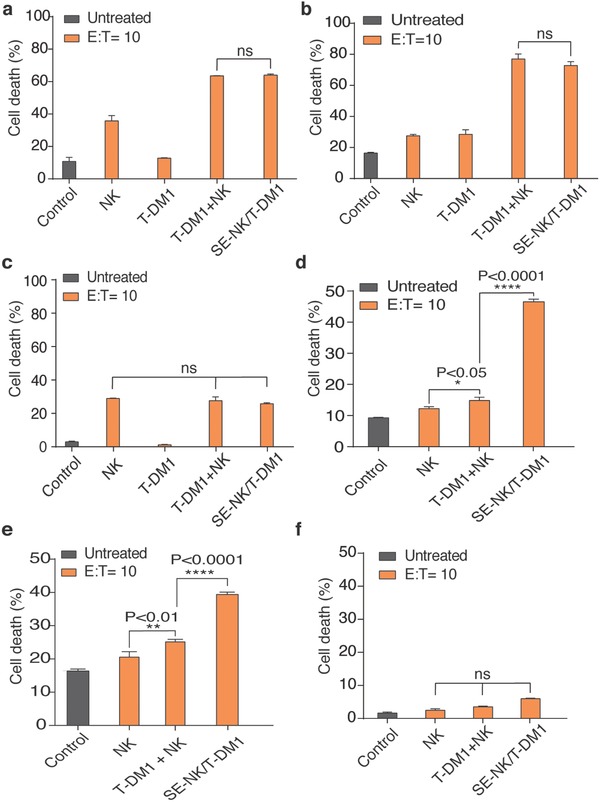
SE‐NK/T‐DM1 cells induce targeted cytotoxicity on HER2‐positive cancer cells. a–c) Cancer cell death induced by coincubating T‐DM1+NK cotreatment or SE‐NK/T‐DM1 cells with SK‐BR‐3 cells, Calu‐3 cells, or MDA‐MB‐231 cells. Cancer cells labeled with CMAC (blue) dye were incubated with unmodified NK cells, T‐DM1, T‐DM1+NK cotreatment, or SE‐NK/T‐DM1 cells for 24 h without removing the unbound NK cells. Similar level of cancer cell death resulted from both T‐DM1+NK cotreatment or SE‐NK/T‐DM1 cells on SK‐BR‐3 cells and Calu‐3 cells in a confined volume. In MDA‐MB‐231 cells, only the anticancer activity of NK cells was observed. d–f) Targeted anticancer activity of SE‐NK/T‐DM1 cells was determined by removing the unbound NK cells after the coincubation of SE‐NK/T‐DM1 cells with SK‐BR‐3 cells, Calu‐3 cells, and MDA‐MB‐231 cells. Cancer cells labeled with CMAC (blue) dye were incubated with T‐DM1+NK cotreatment, SE‐NK/T‐DM1 cells, or other corresponding treatments. Unbound NK cells were removed after 2 h of incubation and the remaining cell mixtures were incubated for additional 24 h. Removal of unbound NK cells allows testing for target cancer homing effect. Enhanced HER2‐targeted anticancer activity was observed with SE‐NK/T‐DM1 cells but negligible cancer cell death was observed in MDA‐MB‐231 cells. Cancer cell death was measured with flow cytometry using an annexin V Alexa Fluor 488 and propidium iodide kit. Data represent mean ± SD (ns, not significant, *P < 0.05, **P < 0.01, ****P < 0.0001, by two‐way ANOVA with Bonferroni post hoc tests).

To validate the therapeutic advantages of SE‐NK/T‐DM1 cells over the T‐DM1+NK cotreatment, we first treated the cancer cells with SE‐NK/T‐DM1 cells and T‐DM1+NK cotreatment for 24 h without the removal of unbound immune cells (**Figure** **4**a,b). Both treatments induced similar levels of cancer cell death, indicating that continuous exposure to the T‐DM1+NK cotreatment allows enough time for NK cells to identify dying cancer cells affected by T‐DM1 in a confined well system. In MDA‐MB‐231 cells, only the anticancer activity of NK cells was observed in both treatment groups (Figure 4c). Subsequently, cancer cells were incubated with the identical treatments for 2 h and the unbound effector cells were removed to mimic the in vivo cancer‐targeted homing effect. We further incubated the remaining cancer‐bound effector cells with the target cells for 24 h and recorded the resulting cancer cell death. In SK‐BR‐3 cells and Calu‐3 cells, we found that the level of cancer cell death induced by SE‐NK/T‐DM1 cells was greater than that induced by NK cell or T‐DM1+NK cotreatment, while no significant cell death was noticed in MDA‐MB‐231 cells (Figure 4d–f). This is due to the fact that a higher number of SE‐NK/T‐DM1 cells remained bound to SK‐BR‐3 cells and Calu‐3 cells, resulting in an augmented level of anticancer activity.

**Figure 5:**
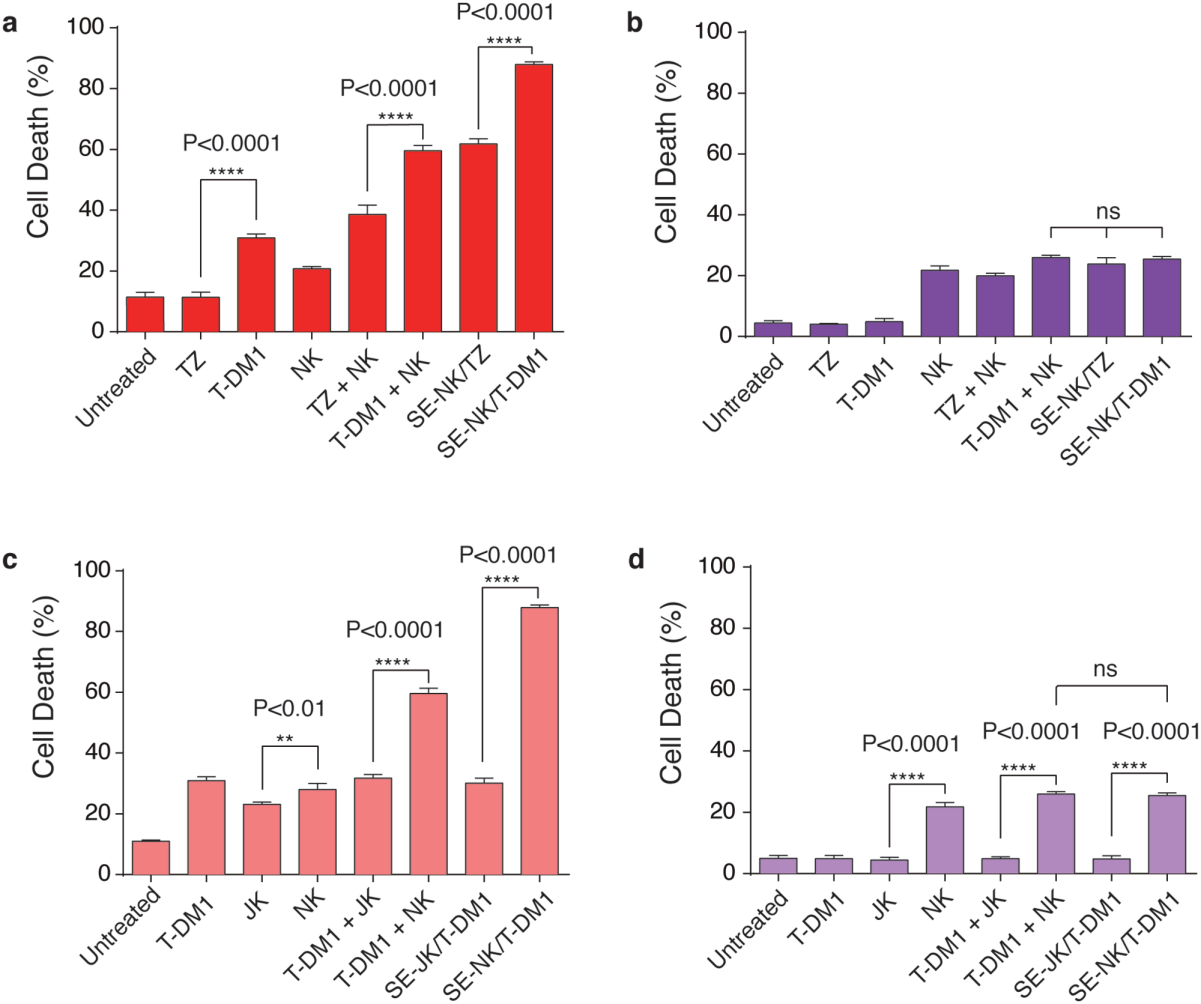
SE‐NK/T‐DM1 cells exert strong anticancer effects against the target cancer cells through combined cytotoxic activities of all components. Effects of T‐DM1 on cancer cell death. The targeted anticancer activity of antibody and chemotherapeutic agents was demonstrated by comparing the cytolytic effects of TZ and T‐DM1, TZ+NK cotreatment and T‐DM1+NK cotreatment, and SE‐NK/TZ cells and SE‐NK/T‐DM1 cells against a) SK‐BR‐3 cells or b) MDA‐MB‐231 cells. Cancer cells labeled with CMAC (blue) were coincubated with each treatment. Unbound immune cells were removed after 2 h and the remaining cell mixtures were incubated for additional 24 h. T‐DM1 induced greater cancer cell death compared to TZ due to the DM1 conjugation. Likewise, SE‐NK/T‐DM1 cells showed enhanced anticancer activity compared to SE‐NK/TZ cells. Surface‐engineered antibody or ADCs on NK cells induced greater cytotoxicity against the target cancer cells compared to unmodified NK cells or corresponding cotreatments. In MDA‐MB‐231 cells, only the nonspecific activity of NK cells was observed. c,d) Identification of the anticancer activity of NK cells. Experimental approach described for (a) and (c) was followed with an inclusion of Jurkat (JK) cells, a surrogate negative T‐cell line. The cancer cell death induced by SE‐NK/T‐DM1 cells was compared to that of SE‐JK/T‐DM1 cells against c) SK‐BR‐3 cells and d) MDA‐MB‐231 cells. JK showed significantly lower cytotoxicity compared to NK cells. SE‐JK/T‐DM1 cells only displayed the anticancer activity of T‐DM1 and SE‐NK/T‐DM1 cells showed superior anticancer activity over all treatment. Cancer cell death was measured with flow cytometry using an annexin V Alexa Fluor 488 and propidium iodide kit. Data represent mean ± SD (ns, not significant, **P < 0.01, ****P < 0.0001, by one‐way ANOVA followed by Tukey post hoc tests).

Next, we assessed the effect of trastuzumab, DM1, and NK cells, contained in SE‐NK/T‐DM1 cells on cancer cell viability. To identify the anticancer effect of DM1, we prepared trastuzumab surface‐engineered NK (SE‐NK/TZ) cells and compared the cancer cell death induced by SE‐NK/TZ cells and SE‐NK/T‐DM1 cells. Cancer cell death was analyzed 24 h of coincubation after the removal of unbound NK cells 2 h after the treatment. As expected, T‐DM1 exhibited a greater cytolytic effect against SK‐BR‐3 cells than TZ (**Figure** **5**a). The resulting enhanced cancer cell death was due to the addition of DM1. NK cells and TZ (TZ+NK) cotreatment showed slightly improved cytotoxicity compared to the NK cells alone however it was much less compared to the T‐DM1+NK cotreatment. The treatments involving T‐DM1 further enhanced anticancer activity against HER2‐positive cancer cells, and SE‐NK/T‐DM1 cells exhibited anticancer activity superior to all other treatments. We postulated that DM1 contained in T‐DM1 induced an increase of ≈20% in the death of HER2‐positive cancer cells. Except for the nonspecific cytolytic activity of NK cells, none of the treatments induced significant cytotoxicity in MDA‐MB‐231 cells (Figure 5b).

We continued to determine the effects of immune effector cells by comparing NK cells to Jurkat (JK) cells, a surrogate negative T‐cell line, in identical experimental settings. Cytotoxicity of T‐DM1 surface‐engineered JK (SE‐JK/T‐DM1) cells and SE‐NK/T‐DM1 cells was tested against SK‐BR‐3 cells and MDA‐MB‐231 cells. As expected, NK cells showed higher cytolytic activity in SK‐BR‐3 cells compared to JK cells (Figure 5c). T‐DM1+NK cotreatment caused nearly 27% more cancer cell death compared to the T‐DM1 with JK cells (T‐DM1+JK) cotreatment. Similarly, SE‐NK/T‐DM1 cells induced ≈58% greater cancer cell death in SK‐BR‐3 cells compared to SE‐JK/T‐DM1 cells. Consistently, no significant differences in cell death beyond the NK cell activity were observed in MDA‐MB‐231 cells (Figure 5d). SE‐NK/T‐DM1 cells did not show augmented cytotoxicity against HER2‐negative MDA‐MB‐231 cells compared to NK cells alone, therefore, the possibility of DMEP‐PEG‐T‐DM1 nonspecifically activating NK cells can be ruled out. These results confirm that the individual components of SE‐NK/T‐DM1 cells are crucial factors in producing the combinatorial anticancer efficacy. Moreover, embedding ADCs in immune cells using our one‐step method enhances the anticancer efficacy beyond ADC alone, immune cells alone, or cotreatment of ADC and immune cells.

To determine whether or not NK cells were activated upon incorporation of DMPE‐PEG‐T‐DM1 on their surface, we assessed the level of CD107a expression, a prominent degranulation marker,^23^, ^24^ on SE‐NK/T‐DM1 cells and unmodified NK cells upon engaging the target cancer cells (Figure S4, Supporting Information). As a positive control, NK cells and SE‐NK/T‐DM1 cells were activated through PMA/Ionomycin stimulation (Figure S4a, Supporting Information). In NK cells and T‐DM1+NK cell cotreatment groups, the levels of CD107a expression stayed at the basal level even when incubated with the cancer cells (Figure S4, Supporting Information). Expression of CD107a was however amplified in SE‐NK/T‐DM1 cells upon contact with the target cancer cells, SK‐BR‐3 cells, and Calu‐3 cells, (Figure S4b,c, Supporting Information). This increase was absent in the nontarget MDA‐MB‐231 cells (Figure S4d, Supporting Information). These results prove the absence of nonspecific activation of NK cells following the surface modification with DMPE‐PEGT‐DM1 and support the target‐specific activation of SE‐NK/T‐DM1 cells.

**Figure 6:**
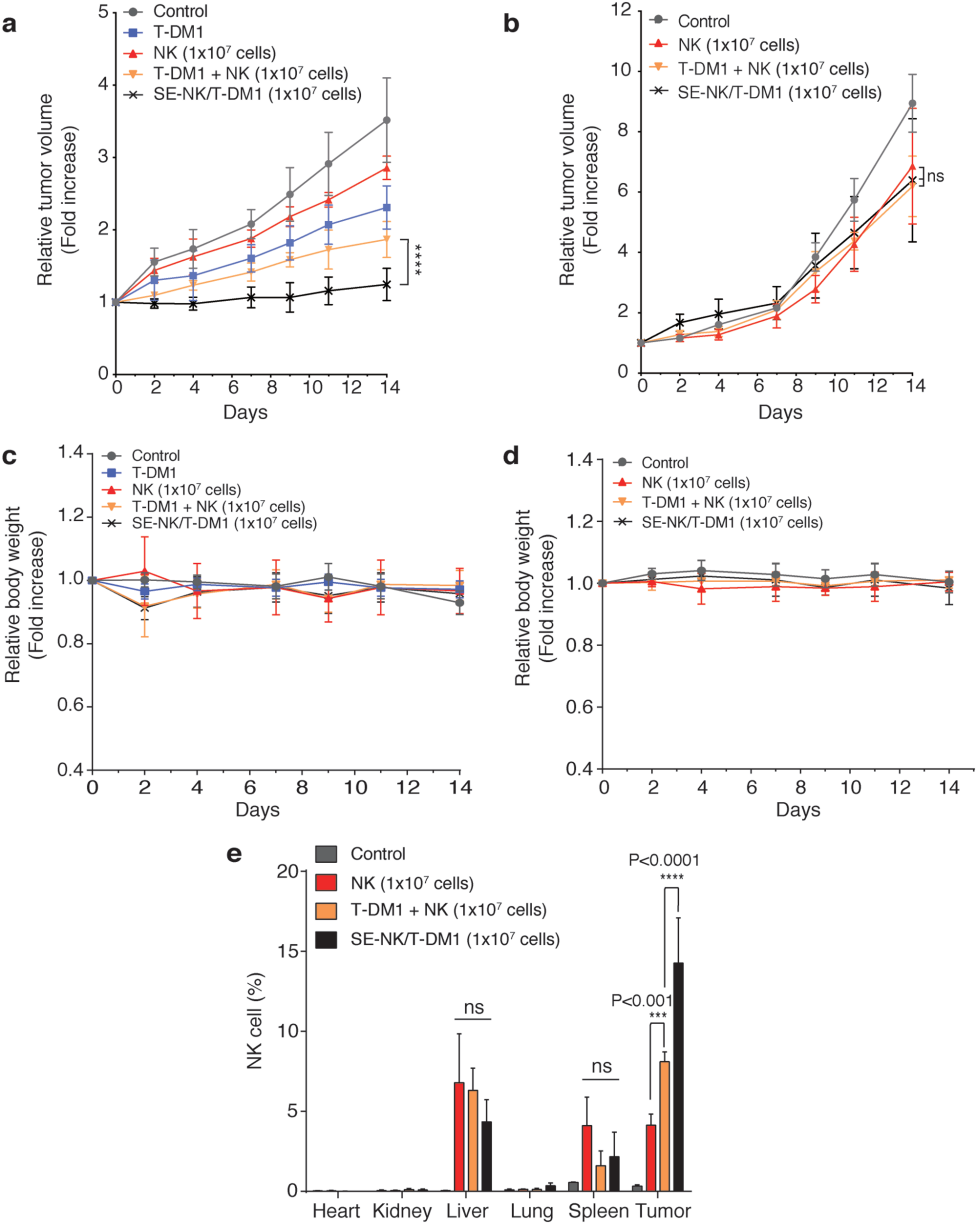
SE‐NK/T‐DM1 cells exhibit superior anticancer efficacy against HER‐2 positive cancer. a,b) Relative tumor volume change of HER2‐positive Calu‐3 cancer or HER2‐negative MDA‐MB‐231 cancer. Tumors were inoculated on the left flank of female NOD *scid* Gamma (NSG) mice. Tumor‐bearing mice (*n* = 4, except for the control group in MDA‐MB‐231 cells that had *n* = 3) received weekly treatment of no treatment, 0.21 mg of T‐DM1, 1 × 10^7^ NK cells, 0.21 mg of T‐DM1+1 × 10^7^ NK cotreatment, or 1 × 10^7^ SE‐NK/T‐DM1 cells through tail vein infusion for 14 d. Compared to T‐DM1+NK cell cotreatment, SE‐NK/T‐DM1 cells significantly suppressed the tumor growth. All agents were freshly prepared in 250 µL of PBS and the infusion was scheduled for 1 min. Data represent mean ± SD (****P < 0.0001, two‐way repeated measure ANOVA with Bonferroni post hoc tests). c,d) Body weight change of Calu‐3 models and MDA‐MB‐231 models. e) Biodistribution of SE‐NK/T‐DM1 cells in Calu‐3 tumor‐bearing NSG mice (*n* = 3). Animals received no treatment, 1 × 10^7^ NK cells, 0.21 mg of T‐DM1+1 × 10^7^ NK cotreatment, or 1 × 10^7^ SE‐NK/T‐DM1 cells through tail vein infusion. All agents were freshly prepared in 250 µL of PBS for 1 min infusion. Tumor and other vital organs were harvested at 24 h post‐treatment. Single‐cell suspension was prepared from the harvest tissues and APC‐conjugated anti‐CD56 antibodies were applied to detect NK cells. Flow cytometer was used to count NK cells among 1 × 10^5^ total cells. Data represent mean ± SD (ns, not significant; ***P < 0.001; ****P < 0.0001; two‐way ANOVA with Bonferroni post hoc tests).

We compared the in vivo anticancer activity of SE‐NK/T‐DM1 cells to that of T‐DM1+NK cotreatment using HER2‐positive Calu‐3 models and HER2‐negative MDA‐MB‐231 models. Tumor‐bearing NOD *scid* Gamma (NSG, NOD.Cg‐*Prkdc^scid^ Il2rg^tm1Wjl^*/SzJ) mice administered with 1 × 10^7^ SE‐NK/T‐DM1 cells received ≈210 µg of T‐DM1, which is similar to the recommended dose found in the literature for mice models (7–10 mg kg^−1^).^25^ In the HER2‐positive tumor model, SE‐NK/T‐DM1 cells exhibited the strongest anticancer efficacy through the combinatorial effects (**Figure** **6**a). The T‐DM1+NK cotreatment inhibited tumor growth when compared to the control group. Treatment of SE‐NK/T‐DM1 cells demonstrated a substantial suppression in tumor growth compared to the T‐DM1+NK cotreatment. In the HER2‐negative tumor model, no significant difference in the tumor growth suppression was observed among all treatment groups (Figure 6b). The Calu‐3 models and MDA‐MB‐231 models had steady body weights during the study period, indicating that the treatments caused no severe toxicity (Figure 6c,d).

For the biodistribution of SE‐NK/T‐DM1 cells in Calu‐3 tumor models, we observed negligible accumulation of NK cells in the heart, kidneys, and lungs (Figure 6e). While NK cells were detected in the liver and spleen, no significant differences in the number of NK cells were observed among the treatment groups. Compared to the unmodified NK cells and T‐DM1+NK cotreatment, a greater number of NK cells were spotted in the tumor tissues that received SE‐NK/T‐DM1 cells. The in vivo experimental results from the efficacy and biodistribution studies confirm that SE‐NK/T‐DM1 cells prepared by our one‐step method selectively migrate toward targeted tumor tissue and induce cancer cell death.

Surface engineering of NK cells with T‐DM1 enabled simultaneous accumulation of T‐DM1 and NK cells in the target tumor tissue. Binding of T‐DM1 to HER2‐positive cancer cells would inhibit the downstream signaling pathway associated with PI3K and AKT and the chemotherapeutic agent, DM1, disrupts the microtubule networks in the target cells, both of which lead to cell cycle arrest and cell apoptosis.^17^, ^26^ SE‐NK/T‐DM1 cells migrated toward the antigen‐expressing cancer cells from physical contact with the target cancer cells, which in turn increased the chance to stimulate the cytolytic function of NK cells. These NK cells in close contact with the target cancer cells, then, eradicate the cancer cell undergoing apoptosis by recognizing damage‐associated molecular patterns (DAMP) expressed on the dying cancer cells.^27^, ^28^, ^29^, ^30^, ^31^

Allogeneic immune cells, the cell‐of‐interest to be weaponized by our one‐step method, gained attention as a suitable solution to reinforce the diminishing active immune cell population in cancer patients due to their low occurrence adverse effects, high tumor‐specific cytotoxicity, predictable anticancer activity, and ease of ex vivo expanding, maintaining, and activating a large cell population.^32^, ^33^, ^34^, ^35^, ^36^ Even the most advanced methods of adoptive immune cell therapy, including chimeric antigen receptor‐T (CAR‐T) cells, still face challenges such as long production time, labor intensity generation process, high cost for widespread application, and limited efficacy in solid tumors.^37^, ^38^, ^39^, ^40^, ^41^, ^42^ Using our one‐step method, however, advanced immune cells with a specific tumor‐homing capability and potent anticancer activity based on chemoimmunotherapy can be generated instantly at the bedside, greatly reducing the time and cost required to obtain sufficient tumor‐reactive immune cells. More importantly, the surface‐engineered immune cells with ADCs generated by our approach would have enhanced efficacy even in solid tumors because antibodies, chemotherapeutic agents, and immune cells, all of which comprise our advanced immune cells, work in concert to eradicate the target cancer. One potential problem of expanding the application of hydrophobized ADCs to other immune cells may be the presence of Fc receptors. Fortunately, the conjugation of DMPE‐PEGs to ADCs may circumvent the issue by masking the Fc region to increase the steric hindrance that lowers the binding affinity of Fc receptors on immune cells.^43^ Other creative antibody engineering methods, such as using single chain variable fragment (scFv) and altering Fc region to reduce Fc receptor binding affinity, can be employed as an alternative strategy for our surface engineering purpose.^44^, ^45^, ^46^

## 3 Conclusion

We demonstrated that our one‐step method constitutes a new platform to produce advanced form of chemoimmunotherapy that can achieve elevated levels of anticancer efficacy in many types of cancers including solid tumors and the product of our innovative mode of chemoimmunotherapy is promising. Furthermore, this approach enables embedding any type of ADCs on the surface of any class of immune cells, including T cells, DCs, and macrophages, in order to transform them to combat a broad spectrum of cancers. The applicability of surface‐engineered immune cells is expected to be high because many new ADCs and new types of allogeneic immune cells currently undergoing discovery and development are viable candidates for our one‐step method to generate advanced chemoimmunotherapy that potentially target different types of cancers. Our one‐step method, a modular design allowing for the matching of allogeneic immune cells and ADCs based on particular needs, will be utilized to produce a wide range of targeted chemoimmunotherapy in the near future that adheres to the goal of creating “off‐the‐shelf” reagents.

## 4 Experimental Section

*Surface Engineering of Immune Cells*: NK cells and JK cells were modified with DMPE‐PEG‐T‐DM1 or DMPE‐PEG‐TZ to generate SE‐NK/T‐DM1 cells, SE‐NK/TZ cells, and SE‐JK/T‐DM1 cells. Briefly, 5 × 10^5^ immune cells were incubated with different amounts of DMPE‐PEG‐T‐DM1 or DMPE‐PEG‐TZ in 100 µL PBS at room temperature for 15 min. After the modification, cells were washed twice with 1 mL PBS. The one‐step method was optimized with 100 µg of DMPE‐PEG‐T‐DM1 per 5 × 10^5^ immune cells.

*Characterization of Surface‐Engineered Cells*: SE‐NK/T‐DM1 cells were prepared with FITC‐labeled DMPE‐PEG‐T‐DM1 according to the procedure described above. SE‐NK/T‐DM1‐FITC cells were visualized by confocal microscopy (Nikon A1R, Nikon; Ex/Em = 495/520 nm). Collected images were processed with ImageJ software. Changes in cell viability and proliferative functions following the modification were determined using CCK‐8. Surface retention times of T‐DM1 on the SE‐NK/T‐DM1 cell membrane were measured using Alexa 488‐conjugated goat anti‐human (H+L) antibodies (Ex/Em = 495/520 nm). SE‐NK/T‐DM1 cells incubated in complete growth media were withdrawn at each time point and labeled with 10 µg of Alexa 488‐conjugated goat anti‐human (H+L) antibodies. Fluorescent signals were measured by flow cytometry and analyzed by FlowJo software. The availability of NK cells receptors after the surface engineering was tested using APC‐conjugated anti‐CD56 antibodies (Ex/Em = 650/660 nm) or APC‐conjugated anti‐2B4 antibodies (Ex/Em = 650/660 nm). Each antibody was applied onto SE‐NK/T‐DM1‐FITC cells according to the manufacturer's recommended amount. The availability of CD56 and 2B4 on SE‐NK/T‐DM1 was detected by flow cytometry and analyzed by FlowJo software.

*Selective Binding, Transfer, and Internalization of T‐DM1*: SK‐BR‐3 cells, Calu‐3 cells, and MDA‐MB‐231 cells were labeled with 2 × 10^−6^ m of CellTracker Red CMTPX (Ex/Em = 577/602 nm). Cancer cells were seeded on 24‐well plate at a density of 4 × 10^4^ cells/well 24 h prior to treatment. NK cells were labeled with 1 × 10^−6^ m of CellTracker Blue CMAC (Ex/Em = 353/466 nm) prior to surface engineering with DMEP‐PEG‐T‐DM1. Cancer cells were coincubated with unmodified NK cells, T‐DM1+NK cotreatment, and SE‐NK/T‐DM1 cells at an *E*:*T* ratio of 10:1. After 30 min, unbound NK cells were removed and all remaining cells were collected. The number of NK cells was quantified per 1 × 10^4^ cancer cells by flow cytometry and the remaining *E*:*T* ratio was calculated.

Transfer of T‐DM1 from SE‐NK/T‐DM1 cells to target cancer cells was examined using confocal microscopy. SK‐BR‐3 cells, Calu‐3 cells, and MDA‐MB‐231 cells labeled with 2 × 10^−6^ m of CellTracker Red CMTPX and seeded on a Lab‐Tek II eight‐chambered cover glass slide at a density of 1 × 10^4^ cells/well 24 h prior to treatment. NK cells labeled with 1 × 10^−6^ m CellTracker Blue CMAC were modified with 100 µg of DMPE‐PEG‐T‐DM1‐FITC. After the modification, 1 × 10^5^ SE‐NK/T‐DM1 cells were coincubated with the cancer cells for 30 min and washed with PBS to remove the unbound effector cells. Coincubated cells were imaged by confocal microscopy and collected images were processed by ImageJ software.

Internalization of T‐DM1 was visualized using a similar procedure. Cancer cells labeled with NucBlue Live ReadyProbe Reagent (Ex/Em = 360/460 nm) were seeded on a Lab‐Tek II eight‐chambered cover glass at a density of 1 × 10^4^ cells/well 24 h prior to treatment. NK cells labeled with 1 × 10^−6^ m CellTracker Red CMTPX were modified with 100 µg of DMPE‐PEG‐T‐DM1‐FITC. Cancer cells were treated with T‐DM1‐FITC or CMPTX‐labeled SE‐NK/T‐DM1‐FITC cells at an *E*:*T* ratio of 10:1. Unbound T‐DM1‐FITC and SE‐NK/T‐DM1‐FITC cells were thoroughly removed after 30 min. Internalization of T‐DM1‐FITC (Ex/Em = 495/520 nm) was imaged using confocal microscopy at the initial time point and 6 h later. Collected images were processed with ImageJ software.

*Cancer‐Targeted Cytotoxicity of SE‐NK/T‐DM1 Cells*: SK‐BR‐3 cells, Calu‐3 cells, and MDA‐MB‐231 cells were labeled with 2 × 10^−6^ m of CellTracker Blue CMAC and seeded at a population of 1 × 10^4^ cells/well on a 48‐well plate 24 h prior to treatment. Cancer cells were coincubated with unmodified JK cells, unmodified NK cells, TZ, T‐DM1, T‐DM1+JK cotreatment, T‐DM1+NK cotreatment, SE‐NK/T‐DM1 cells, SE‐NK/TZ cells, or SE‐JK/T‐DM1 cells at an *E*:*T* ratio of 10:1 in 600 µL of complete media. Cancer cells receiving T‐DM1 treatment received 2.1 µg of T‐DM1 that corresponds to the T‐DM1 amount on SE‐NK/T‐DM1 treated at an *E*:*T* ratio of 10:1. All treatments were washed 2 h after the coculture, and the remaining cancer‐bound effector cells were further incubated for 24 h. All cells were harvested and labeled with the Annexin V Alexa Fluor 488 and propidium iodide kit (Annexin Ex/Em = 495/520 nm and propidium Ex/Em = 535/617) after 24 h of coincubation. Cancer cell death was analyzed by flow cytometry.

To distinguish the effects of antibodies, chemotherapeutic agents, and immune cells, cancer cells labeled with CMAC were coincubated with SE‐NK/T‐DM1 cells, SE‐NK/TZ cells, SE‐JK/T‐DM1 cells, or other corresponding treatments at *E*:*T* ratio of 10:1 in 600 µL of complete media. Unbound effector cells were removed 2 h after the initial coincubation and the remaining cell mixtures were further incubated for 24 h. Cancer cells receiving T‐DM1 treatment received 2.1 µg of T‐DM1 that corresponds to the T‐DM1 amount on surface‐engineered immune cells with ADCs treated at an *E*:*T* ratio of 10:1. Resulting cancer cell death was identified with Annexin V Alexa Fluor 488 and propidium iodide kit (Annexin Ex/Em = 495/520 nm and propidium Ex/Em = 535/617) using flow cytometry and analyzed by FlowJo software.

*In Vivo Tumor Efficacy and Biodistribution*: In vivo studies were conducted with six‐week‐old female NOD *scid* gamma (NSG, NOD.Cg‐*Prkdc^scid^ Il2rg^tm1Wjl^*/SzJ) mice purchased from the Jackson Laboratory (Bar Harbor, ME). Each mouse was subcutaneously inoculated with 1 × 10^7^ cells of Calu‐3 cells or MDA‐MB‐231 cells on the left flank. Cancer cells were suspended in PBS supplemented with 10% (v/v) Matrigel (Fisher Scientific, Bedford, MA). Tumor volume was recorded three times per week by measuring the length and the width of the tumor with a caliper and calculating the tumor volume on the basis of the following formula: *V* = 0.5*ab* ^2^, using the longest (*a*) and shortest (*b*) diameters of the tumor. When the tumor volume reached ≈100 mm^3^, tumor‐inoculated mice were randomly assigned to the experimental groups. Control group (*n* = 4 for Calu‐3 model, *n* = 3 for MDA‐MB‐231 model) received no treatment but the study groups (*n* = 4 per group) were weekly administrated with 0.21 mg of T‐DM1, 1 × 10^7^ NK cells, 0.21 mg of T‐DM1+1 × 10^7^ NK cotreatment, or 1 × 10^7^ SE‐NK/T‐DM1 cells through tail vein infusion for two weeks (Day 0 and Day 7). All agents were freshly prepared in 250 µL of PBS and the infusion was completed in 1 min. Tumor growth and body weight were monitored for 14 d and relative tumor volume was calculated by dividing the recorded volume with the initial volume.

For biodistribution, NSG mice‐bearing Calu‐3 tumors (*n* = 3) received no treatment, 1 × 10^7^ NK cells, 0.21 mg of T‐DM1+1 × 10^7^ NK cotreatment, or 1 × 10^7^ SE‐NK/T‐DM1 cells through tail vein infusion. All agents were freshly prepared in 250 µL of PBS for 1 min infusion. Tumor and major organs, including heart, kidneys, liver, lungs, and spleen, were harvested 24 h after the treatment. Single‐cell suspension of each harvested organ was prepared using the gentleMACS Dissociator and tissue dissociation kits (Miltenyi Biotec, Bergisch Gladbach, Germany) following the instructions provided by the manufacturer. Half of each cell mixture was incubated with 30 µg of an APC‐conjugated anti‐CD56 (Ex/Em = 650/660 nm) antibody for 1 h at 4 °C. Resulting cell mixtures were washed twice with cold PBS and the presence of NK cells were detected from counting 1 × 10^5^ total cells by flow cytometry. Collected results were analyzed by FlowJo.

*Statistical Analysis*: Statistical analysis was performed in Graphpad Prism 6. All data are presented in mean ± SD. All data were analyzed with one‐way ANOVA with Bonferroni post hoc tests or two‐way ANOVA with Bonferroni post hoc tests. Statistical significance threshold of each test was set at *P* < 0.05: ns = not significant, *P* > 0.05; *, *P* < 0.05; **, *P* < 0.01; ***, *P* < 0.001; ****, *P* < 0.0001.

*Animal Ethics*: All animal experiments were approved by the University of Utah Institutional Animal Care and Use Committee. Described animal procedures were conducted according to guidelines and regulations.

## Conflict of Interest

The authors declare no conflict of interest.

## Supporting information

Supplementary
