# Supplementary material for: One‐Step Method for Instant Generation of Advanced Allogeneic NK Cells
Source: Adv Sci (Weinh). 2018 Sep 27;5(11):1800447. doi: 10.1002/advs.201800447 (PMC6247049; doi:10.1002/advs.201800447)
Supplement: Supplementary file 1 — Supplementary [file ADVS-5-1800447-s001.pdf]

Copyright WILEY-VCH Verlag GmbH & Co. KGaA, 69469 Weinheim, Germany, 2018.

ADVANCED SCIENCE Open Access

# Supporting Information

for *Adv. Sci.,* DOI: 10.1002/advs.201800447

# One-Step Method for Instant Generation of Advanced Allogeneic NK Cells

*Daniel Y. Lee, Kwang Suk Lim, Gabriel M. Valencia, Minjin Jung, David A. Bull,* and Young-Wook Won**

Copyright WILEY-VCH Verlag GmbH & Co. KGaA, 69469 Weinheim, Germany, 2018.

# Supporting Information

## One-step Method for Instant Generation of Advanced Allogeneic NK Cells

**Authors:** Daniel Y. Lee[1,2], Kwang Suk Lim[1], Gabriel M. Valencia[1], Minjin Jung[2], David A. Bull[1,2,]*, Young-Wook Won[1,2,]*

## Materials

Trastuzumab (Herceptin®) and ado-trastuzumab emtansine (Kadcyla®) were purchased from Genentech (San Francisco, CA). pVITRO1-Trastuzumab-IgG1/κ was a gift from Andrew Beavil (Addgene plasmid # 61883)[36]. Succinimidyl 4-(N-maleimidomethyl)cyclohexane-1-carboxylate linked DM1 (SMCC-DM1) was acquired from MedKoo Bioscience (Morrisville, NC). 1,2-Dimyristoyl-sn-glycero-3-phosphoethanolamine-poly(ethylene glycol)-NHS (DMPE-PEG-NHS, MW= 5 kDa) was purchased from Nanocs (New York, NY). All other materials were purchased from Thermo Fisher Scientific (Waltham, MA). All cancer cell lines and NK92 cells were purchased from ATCC (Manassas, VA). X-VIVO 15 and IL-2 were purchased from Lonza (Walkersville, MD) and Peprotech (Rocky Hill, NJ), respectively. Cell counting kit (CCK-8) was obtained from Dojindo Molecular Technologies (Kumamoto, Japan). Cell activation cocktail, brefeldin A, and monensin were acquired from Biolegend (San Diego, CA). All other cell culture products were purchased from Thermo Fisher Scientific (Waltham, MA). All antibodies were acquired from Miltenyi Biotec (Bergisch Gladbach, Germany), except for the Alexa 488-conjugated goat anti-human IgG (H+L) antibody (Thermo Fisher). Human IgG total ELISA kit was acquired from eBioscience (San Diego, CA) and R&D Systems (Minneapolis, MN), respectively. Matrigel™ Matrix HC was obtained from Fisher Scientific (Bedford, MA).

Tissue dissociation kit, gentleMACS Dissociator, and associated materials were purchased from Miltenyi Biotec (Bergisch Gladbach, Germany).

**Cell culture**

Human SK-BR-3 and MDA-MB-231 breast cancer cells were maintained in McCoy's 5A media and RPMI 1640 media, respectively. Human Calu-3 lung cancer cells were maintained with RPMI 1640 media. Jurkat cells were grown in RPMI 1640. Cancer cell and Jurkat cell media were supplemented with 10% FBS, 100 U/mL penicillin, and 100 μg/mL streptomycin. Human NK92 cells were maintained in X-VIVO 15 media, containing 500 U/mL IL-2, 10% FBS, 100 U/mL penicillin, and 100 μg/mL streptomycin.

**Generation of recombinant trastuzumab**

Freestyle™ 293-F cells were transfected with pVITRO1-Trastuzumab-IgG1/K under the conditions specified by the Expi293 expression kit (Thermo Fisher Scientific). Transfected cells were grown over 72 hours and the culture media containing the secreted proteins was collected. The culture media was dialyzed against PBS for 24 hours, passed through 0.25 μm filters and purified by FPLC (NGC Scout™ System, Bio-Rad, Hercules, CA) equipped with a protein G affinity column (Thermo Fisher Scientific). Purified trastuzumab (TZ) was dialyzed against PBS using a Slide-A-Lyzer G2 dialysis cassette (MWCO 10 kDa) and stored at -80°C until use.

**Synthesis of T-DM1**

T-DM1 was prepared by conjugating SMCC-DM1 to TZ. SMCC-DM1 was dissolved in DMSO at the concentration of 2 mg/mL and 5-, 10-, or 15-molar excess of SMCC-DM1 was added to 2 mg of TZ dissolved in PBS. The reaction mixture was stirred for 2 hours at room temperature. Unconjugated SMCC-DM1 was removed by overnight dialysis (MWCO

10 kDa) against PBS at 4°C. The resulting T-DM1 was further purified by FPLC using the protein G affinity column followed by additional dialysis (MWCO 10 kDa). Samples of T-DM1 were submitted to the University of Utah Mass Spectrometry & Proteomics Core and the drug-to-antibody ratio of each T-DM1 was calculated from ESI/MS spectrum.

**Antibody activity**

HER2-binding was determined for TZ and T-DM1 synthesized with different molar ratios. All antibodies and ADCs were labeled with FITC. SK-BR-3 cells, Calu-3 cells and MDA-MB-231 cells were incubated with 5 μg of FITC-conjugated antibodies and ADCs including Herceptin® and Kadcyla® and fluorescent signal was measured by using a FACS Canto™ (BD Bioscience). Collected data was analyzed by FlowJo software.

**Cytotoxicity of T-DM1**

Cytotoxicity of the synthesized T-DM1 was compared to Kadcyla®. HER2-positive SK-BR-3 cells, Calu-3 cells and HER2-negative MDA-MB-231 cells, $1\times10^4$ cells/well, was seeded on a 48-well plate and serially diluted T-DM1 or Kadcyla® was added to the media. After 48-hour incubation, cancer cell death was analyzed by an MTT assay. Data were analyzed with GraphPad Prism 6 software using a four-parameter logistic non-linear regression model. Subsequent studies will employ T-DM1 prepared with molar ratio of 10.

**Synthesis of DMPE-T-DM1 and DMEP-TZ**

DMPE-PEGT-DM1 or DMPE-PEG-TZ was prepared by mixing 2 mg of T-DM1 or TZ dissolved in PBS with 15-molar excess of DMPE-PEG-NHS dissolved in DMSO. The reaction mixture was stirred at room temperature for 2 hours. Unconjugated DMPE-PEGs were removed via overnight dialysis (MWCO: 20 kDa) against PBS at 4°C.

**Determination of T-DM1 amount embedded on the NK cell membrane**

SE-NK/T-DM1 cells were prepared by mixing $5\times10^5$ NK cells with 50 μg, 100 μg, or 200 μg of DMPE-PEG-T-DM1. From these preparations, $1\times10^5$ SE-NK/T-DM1 cells were lysed to release T-DM1 from the cell membrane. Cell lysates were applied on Human IgG total ELISA kit (eBioscience, San Diego, CA) for T-DM1 quantification.

**Analysis of NK cell activation**

Expression of CD107a was detected by co-incubating the effector cells with SK-BR-3 cells, Calu-3 cells, or MDA-MB-231 cells. Cancer cells were seeded on a 96-well plate at a density of $2\times10^4$ cells/well. Subsequently, SE-NK/T-DM1 cells, NK cells and T-DM1+NK co-treatment were added to each well at an E:T ratio of 10:1. Unmodified NK cells—stimulated with cell activation cocktail containing phorbol-12-myristate-13-acetate (PMA) and ionomycin—were included as a positive control. Anti-CD107a-FITC antibodies (Clone: H4A3), 10 μg, were added directly into each well. After the cells were incubated at 37°C for 1 hour, brefeldin A and monensin were added to each well according to the manufacturer’s protocol and incubated for an additional 5 hours. All cells were collected, stained with 10 μg anti-CD56-APC antibodies (clone: REA196) to identify NK cells, and examined by flow cytometry. Collected results were analyzed with FlowJo software.

**Reference**

[1] T. S. Dodev, P. Karagiannis, A. E. Gilbert, D. H. Josephs, H. Bowen, L. K. James, H. J. Bax, R. Beavil, M. O. Pang, H. J. Gould, S. N. Karagiannis, A. J. Beavil, Sci Rep 2014, 4, 5885.

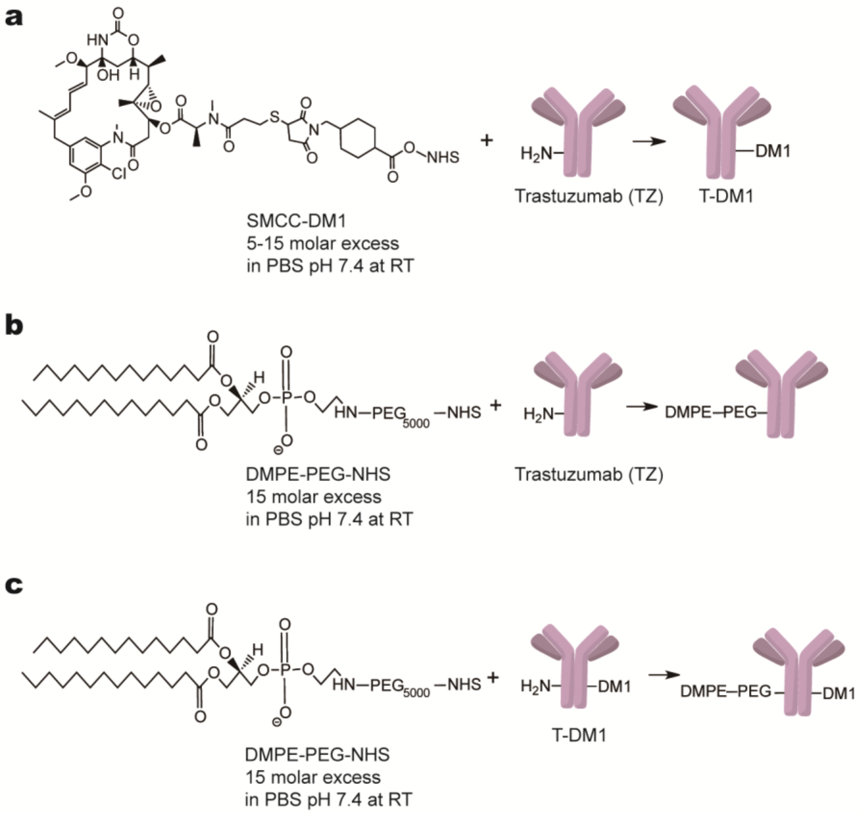


**Figure S1.** Schematic illustration showing the conjugation chemistry to generate (**a**) T-DM1, (**b**) DMPE-PEG-TZ, and (**c**) DMPE-PEG-T-DM1.

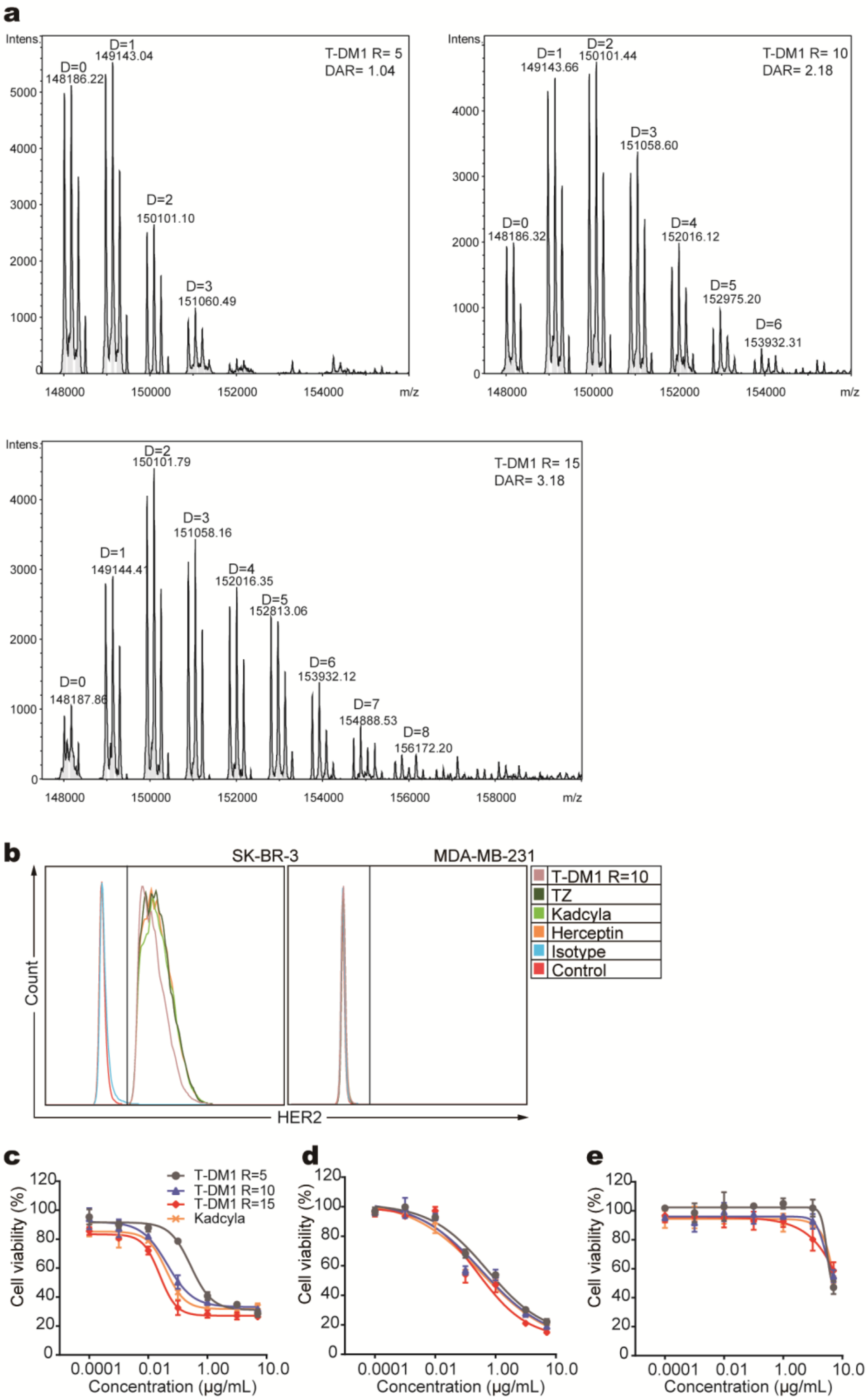


**Figure S2.** Characteristics of synthesized T-DM1. a) ESI/MS spectra of T-DM1 synthesized with different molar ratios of SMCC-DM1. Drug-to-antibody ratio (DAR) was calculated from dividing the sum of all products of area under each peak and the number of drug

conjugated to each species by total area. b) Antigen-specific binding of T-DM1. Antibody activities of TZ, T-DM1 (R=10), Herceptin®, and Kadcyla® were compared in HER2-positive SK-BR-3 cells or HER2-negative MDA-MB-231 cells. Labeled cells were analyzed by flow cytometry. c-e) Cytotoxicity of T-DM1 synthesized with different ratios of SMCC-DM1 and Kadcyla® were tested in (c) SK-BR-3 cells, (d) Calu-3 cells, and (e) MDA-MB-231 cells. Cancer cell death was measured at 48 hours after treatment. T-DM1 (R=10), demonstrating similar cytotoxicity to Kadcyla®, was selected for subsequent surface engineering experiments. Data represent mean ± SD.

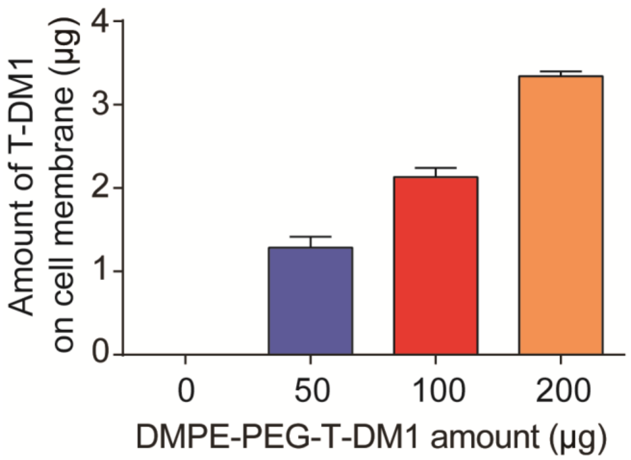


**Figure S3.** T-DM1 amount on the surface of SE-NK/T-DM1 cells. SE-NK/T-DM1 cells were prepared by modifying 5×10$^5$ NK cells with 50 μg, 100 μg, or 200 μg of DMPE-PEG-T-DM1. Then, 1×10$^5$ cells were lysed from each preparation of SE-NK/T-DM1 cells and the membrane fraction was applied to Human IgG total ELISA kit. The amount of T-DM1 on the surface of 1×10$^5$ SE-NK/T-DM1 cells generated with 50 μg, 100 μg, or 200 μg of DMPE-PEG-T-DM1 was 1.2 μg, 2.1 μg, or 3.3 μg, respectively. Therefore, the amount of DMPE-PEG-T-DM1 on the surface of 5×10$^5$ SE-NK/T-DM1 cells generated by each preparation was 6 μg, 10.5 μg, or 16.5 μg, respectively. Data represent mean ± SD.

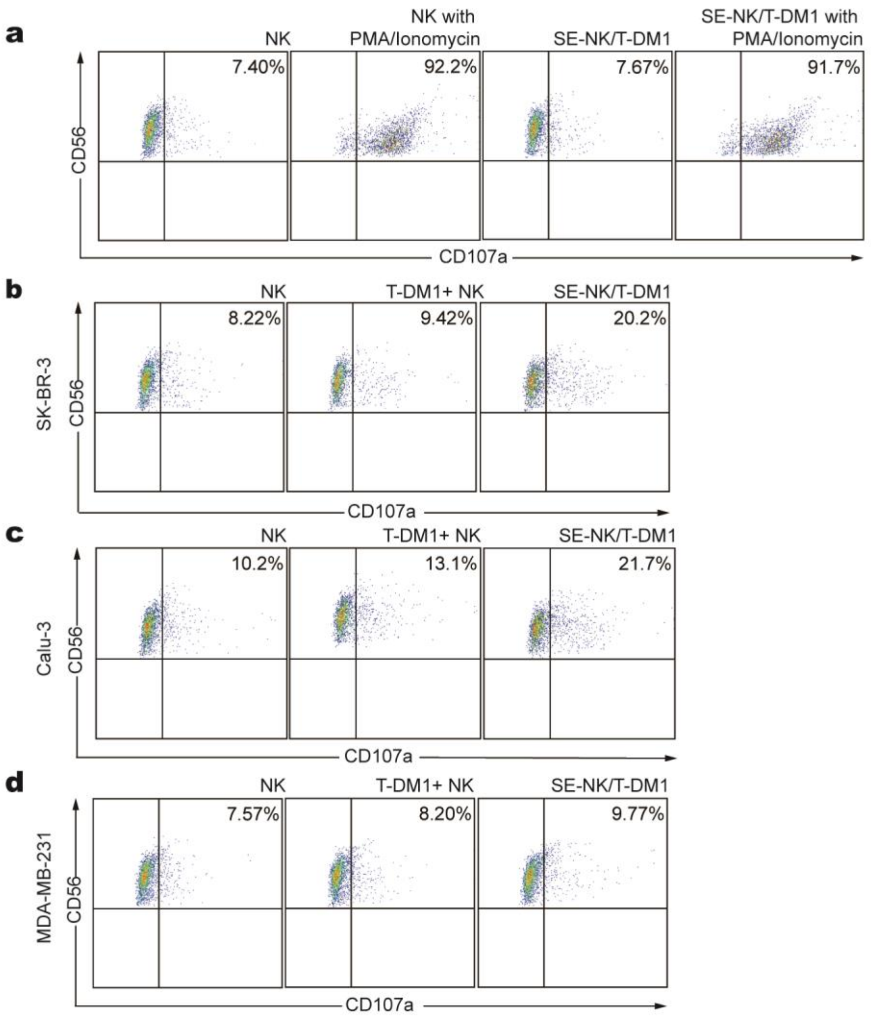


**Figure S4. Analysis of NK cell activation.** Expression of CD107a, a degranulation marker, upon the co-incubation of SE-NK/T-DM1 cells or unmodified NK cells with different cancer cells was analyzed by flow cytometry. (a) CD107a expression in unmodified NK cells or SE-NK/T-DM1 cells grown in culture condition and under PMA/Ionomycin stimulation. CD107a expression on NK cells, T-DM1+NK co-treatment, or SE-NK/T-DM1 cells during the co-incubation with (b) SK-BR-3 cells, (c) Calu-3 cells, and (d) MDA-MB-231 cells. Plot is from one of three independent experiments.
